# STAT3 Activation Promotes Oncolytic HSV1 Replication in Glioma Cells

**DOI:** 10.1371/journal.pone.0071932

**Published:** 2013-08-02

**Authors:** Kazuo Okemoto, Benjamin Wagner, Hans Meisen, Amy Haseley, Balveen Kaur, Ennio Antonio Chiocca

**Affiliations:** 1 Dardinger Center for Neuro-Oncology and Neurosciences, Department of Neurological Surgery, James Cancer Hospital/Solove Research Institute/Comprehensive Cancer Center and Wexner Medical Center, the Ohio State University, Columbus, Ohio, United States of America; 2 Harvey Cushing Neuro-Oncology Laboratories, Department of Neurosurgery, Institute for the Neurosciences at the Brigham, Brigham and Women’s/Faulkner Hospital and Center for Neuro-Oncology, Dana-Farber Cancer Institute, Boston, Massachusetts, United States of America; University of Chicago, United States of America

## Abstract

Recent studies report that STAT3 signaling is a master regulator of mesenchymal transformation of gliomas and that STAT3 modulated genes are highly expressed in the mesenchymal transcriptome of gliomas. A currently studied experimental treatment for gliomas consists of intratumoral injection of oncolytic viruses (OV), such as oncolytic herpes simplex virus type 1 (oHSV). We have described one particular oHSV (rQNestin34.5) that exhibits potent anti-glioma activity in animal models. Here, we hypothesized that alterations in STAT3 signaling in glioma cells may affect the replicative ability of rQNestin34.5. In fact, human U251 glioma cells engineered to either over-express STAT3 or with genetic down-regulation of STAT3 supported oHSV replication to a significantly higher or lesser degree, respectively, when compared to controls. Administration of pharmacologic agents that increase STAT3 phosphorylation/activation (Valproic Acid) or increase STAT3 levels (Interleukin 6) also significantly enhanced oHSV replication. Instead, administration of inhibitors of STAT3 phosphorylation/activation (LLL12) significantly reduced oHSV replication. STAT3 led to a reduction in interferon signaling in oHSV infected cells and inhibition of interferon signaling abolished the effect of STAT3 on oHSV replication. These data thus indicate that STAT3 signaling in malignant gliomas enhances oHSV replication, likely by inhibiting the interferon response in infected glioma cells, thus suggesting avenues for possible potentiation of oncolytic virotherapy.

## Introduction

Malignant gliomas remain formidable cancers to treat and to palliate with conventional standard of care that includes neurosurgery, radiation and chemotherapy. Over the years, several new therapeutic approaches have been attempted including the use of oncolytic viruses (OVs) [1] [2] [3]. These are naturally occurring or genetically engineered recombinant viruses that replicate in a relatively selective fashion in tumor cells. Several clinical trials of OVs have and are being conducted, including trials for malignant gliomas (MG) [4,5]. These have shown the safety of the approach, although efficacy has not been formally evaluated in a phase III clinical trial setting yet.

One of the tenets of OV therapy of tumors is that viral replication and lysis of tumor cells with subsequent distribution/spread to other tumor cells is required for subsequent biologic effects, such as direct cytotoxicity and/or antitumor immunity. Recent trials for systemic cancers have shown evidence for viral replication in tumors. One of the OVs, studied relatively extensively in gliomas, has been based on herpes simplex virus type 1 (HSV-1) [6], [7]. Several different engineered types of this OV have been tested in animal models and in one phase I clinical trial for MG there was reported evidence of a modest degree of replication of the oncolytic HSV (oHSV) in post-injection biopsies [7] [8]. Therefore, attempts in the laboratory to improve OV and oHSV replication have been the focus of recent research, including generating more potent OVs, combining OVs with pharmacologic modulators or immunomodulators, and understanding the tumor host response and barriers to OV efficacy in order to devise avenues to circumvent these.

In this context, recent evidence has emerged to show that activation of the STAT3 pathway represents a central hub in glioma progression and maintenance [9]. STAT3 has been shown to modulate interferon pathways and is thought to be possibly involved in the maintenance of an immune suppressive microenvironment in the MG [10]. Interestingly, there is no published information related to how STAT3 activation would alter wild type (WT) HSV1 or oHSV replication in gliomas. Based on this, we hypothesized that STAT3 activation in MG may actually enhance replication of oHSV in glioma cells through its ability to modulate and down-regulate interferon responses. In this report, we show that genetic or pharmacologic activation of STAT3 in glioma cells enhanced oHSV replication while knockdown or pharmacologic inhibition of STAT3 reduced oHSV replication. As expected, STAT3 activation was associated with a reduction in type 1 interferon signaling in response to the viral infection. Therefore, these results imply that STAT3 activation in MG may be a marker of oHSV efficacy.

## Materials and Methods

### Reagents

Dulbecco’s modified minimal essential medium (DMEM), Neurobasal medium, Hank’s Balanced Salt Solution (HBSS), penicillin and 100 microg/ml streptomycin, GlutaMax, B27 supplement were purchased from Invitrogen (Carlsbard, CA, USA). Human basic fibroblast growth factor (hEGF) and epidermal growth factor (hFGF) were purchased from R&D Systems, Inc (Minneapolis, MN, USA). hIFNalpha1 and human inerleukin-6 (hIL-6) were purchased from Cell Signaling Technology Inc. (Danvers, MA). VPA was purchased from SIGMA-Aldrich (St Louis, MO, USA). Cell proliferation Kit I (MTT) was purchased from Rosh (Indianapolis, IN, USA). LLL12 was a gift from Dr. Lin (Center for Childhood Cancer, The Research Institute at Nationwide Children’s Hospital, Department of Pediatrics, College of Medicine).

### Cells

The human U251 glioma cell line was originally purchased from Sigma-Aldrich (Cat no. 09063001; Sr. Louis, MO) Human U251 glioma cell lines were maintained in DMEM supplemented with 2% fetal bovine serum, 100 U/ml penicillin and 100 microg/ml streptomycin. Human primary OG02 gliomas were previously described as OHG02 [11]. They were harvested under an approved Institutional Review Board (IRB) protocol of from the office of Responsible Research Practices at the Ohio State University Medical Center (Columbus, OH) where consent for collection of surgical specimens was obtained by patients and stored in a tumor tissue bank in a de-identified fashion. They were harvested from a glioblastoma and was grown under conditions that enrich for glioma “stem-like” cells, as neurospheres in Neurobasal medium with x1 GlutaMax (Invitrogen) with B27 supplement (1Å~; Invitrogen), hFGF (20 ng/ml), epidermal growth factor (EGF; 20 ng/ml), penicillin (100 U/ml), streptomycin (100 microg/ml) [11]. All cells were cultured at 37 °C in an atmosphere containing 5% carbon dioxide. pRC/CMV-STAT3-FLAG, pcDNA3-STAT3-HA, pcRC/CMV-FLAG, and pGL4.32-Luc2P/NF-kB-RE/Hygro (Promega, Madison, WI) expression vectors were stably transfected into U251 cells using Cell Line Nucleofector Kit T (Lonza Walkersville Inc., Walkersville, MD). Cignal Lenti STAT3 Reporter (luc)/Puro (SABiosciences, Valencia, CA), shRNAi MISSION Non-Target shRNA Control/Puro (SIGMA-Aldrich), and shRNA STAT3/Puro (SIGMA-Aldrich) lentiviral vectors were used to infect U251 cells, followed by subcloning of stably expressing cells.

### Virus and Virus replication assay

rQNestin34.5 was engineered on a HSV-1 strain F background and engineering of rQNestin34.5 has been reported previously [6]. Viruses were grown, purified and titered on Vero cells. Virus replication assay were performed as published [11]. After incubation with VPA for 20 hours, cells were seeded into 24-well plates at 2 × 10^4^ cells/well in 500 microl. of media and infected with HSV at a multiplicity of infection (MOI) of 0.05. GFP expression was used as an indicator for infection of cells with rQNestin34.4. After 3 days, cells were harvested with supernatants at indicated times in triplicate. After three freeze/thaw cycles and sonication, titers of infectious progeny virus were determined by plaque assay on Vero cells.

### Western blot analysis

Lysates were prepared from U251 cells and U251 stable transfectants by suspension in ice-cold radioimmune precipitation lysis buffer containing 1× phosphate-buffered saline, 1% Nonidet P-40, 0.5% sodium deoxycholate, 0.1% SDS with a fresh addition of 1× protease inhibitor mixture mix (0.15–0.3% trypsin inhibitor units/ml aprotinin, 1 mM sodium orthovanadate, 1 mM benzamidine, 1 microg/ml pepstatin, 10 microg/ml phenylmethylsulfonyl fluoride, and 2 microg/ml leupeptin). The suspension was sonicated and the mixture was incubated for another 20min on ice and finally centrifuged at 10,000 × g for 10 min. The supernatant was saved as total cell lysate, aliquoted, and stored at −80 °C. Nuclear Extracts were prepared from U251 cells by aspirating media, washing cells with 5 ml ice-cold PBS/Phosphatase Inhibitors (Active Motif, Carlsbad, CA) and, after removing the supernatant again, adding 3 mls. of ice-cold PBS/Phosphatase Inhibitors. After removing cells from the dish by gently scraping with a cell lifter, cells were transferred to a pre-chilled 15 ml conical tube. After centrifugation of the cell suspension for 5 minutes at 500 rpm in a centrifuge pre-cooled at 4C, the supernatant was discarded. The cell pellet was saved on ice, before re-suspension in 50 microl of Complete Lysis Buffer (Active Motif) with a couple of vigorous cycles of pipetting. The lysate was vortexed for 10 seconds at the highest setting. After incubating the suspension for 30 minutes on ice on a rocking platform set at 150 rpm, it was vortexed again for 30 seconds at the highest setting. Centrifugation for 10 minutes at 14,000 x g in a microcentrifuge pre-cooled to 4C was performed and then the supernatant (nuclear fraction) was transferred into a pre-chilled microcentrifuge tube. After aliquoting it, it was stored at -80C. Protein (^~^50 microg) was subjected to SDS-PAGE on a 4-12% minigel and transferred to an Immobilon™-P transfer membrane. The membrane was blocked with 5% bovine serum albumin in TBST (10 mM Tris-HCl, pH 8.0, 150 mM NaCl, 0.05% Tween 20) for 1h and incubated with the following antibodies overnight in the cold room; anti-phospho-STAT3 (Tyr705), anti-STAT3 (124H6) Mouse mAb, anti-GAPDH (14C10), anti-NF-kB2 p100/p52 (18D10), anti-Histone H3, anti-HA-Tag (6E2), anti-DYKDDDDK Tag (anti-FLAG) were purchased from Cell Signaling Technology Inc. Proteins were visualized by chemiluminescence with SuperSignal West Pico (Thermo Fisher Scientific Inc., Rockford, IL) and exposed to x-ray film.

### Quantitative RT-PCR

Total RNA was isolated using Quick-RNA miniprep kit (Zymo Reserch Inc., Irvine, CA) and reverse transcribed using the ImProm-II Reverse Transcriptase (Promega). Quantitative real-time PCR was performed using a Replex2 Master Cycler (Eppendorf, Hauppauge, NY) and Power SYBR Green PCR Master Mix (Applied Biosystems, Carlsbard, CA). The following sequences of PCR primers were used for the analysis: IFNα; F acccacagcctggataacag and R actggttgccatcaaactcc, IFNB; F actgcctcaaggacaggatg and R tgctgcagctgcttaatctc, TNFA; F ccgtctcctaccagaccaag and R ggaagacccctcccagatag, gC；F ccttgccgtggtcctgtgga and R gttggggtttggggtcgatg, PKR; F atgatggaaagcgaacaagg, R cagcaagaattagccccaaa STAT1; F tgatggccctaaaggaactg R cagagcccactatccgagac, STAT3, F cctggtgtctccactggtct, R ggcaggtcaatggtattgct, IRF3: F aggaccctcacgacccacataa, R ggccaacaccatgttacccagt, IRF7; ccacgctataccatctacctgg, R ccataaggaagcactcgatgtc, GAPDH; F ggagtcaacggatttggtcg, R ggaatcattggaacatgtaaacc.

### Luciferase activity

 Luciferase activity was analyzed at 8 hours or at indicated times post infection of oHSV using the Luciferase Reporter Assay System (Promega), following the manufacturer’s instructions.

### Cell viability assay by MTT exclusion assay

Cell were treated with VPA for 20h the before infection with rQNestin34.5. Three days after infection, cell viability was determined by MTT assay (Rosh) following the instruction manual. Triplicate wells were counted each time.

### Live cell imaging

50,000 U251 cells labeled with the RFP cell tracker dye (Invitrogen) were plated in 500 microl of medium overnight at 37° C. The following day, the cells were treated concurrently with 0.5 microM LLL12 and rQNestin34.5 at an MOI of 0.1 in 500 microl of medium. The cells were imaged 24 hours later. Virus replication/expression was quantified via determining the percent of GFP positive cells/well. We assayed n=3 per treatment group. Three random fields were counted/ well (20x). A two-tailed, student’s T-Test was used to determine statistical significance, with p<0.05 considered as statistically significant.

## Results

### STAT3 activation enhances oHSV replication in a glioma cell line

**Figure 1:**
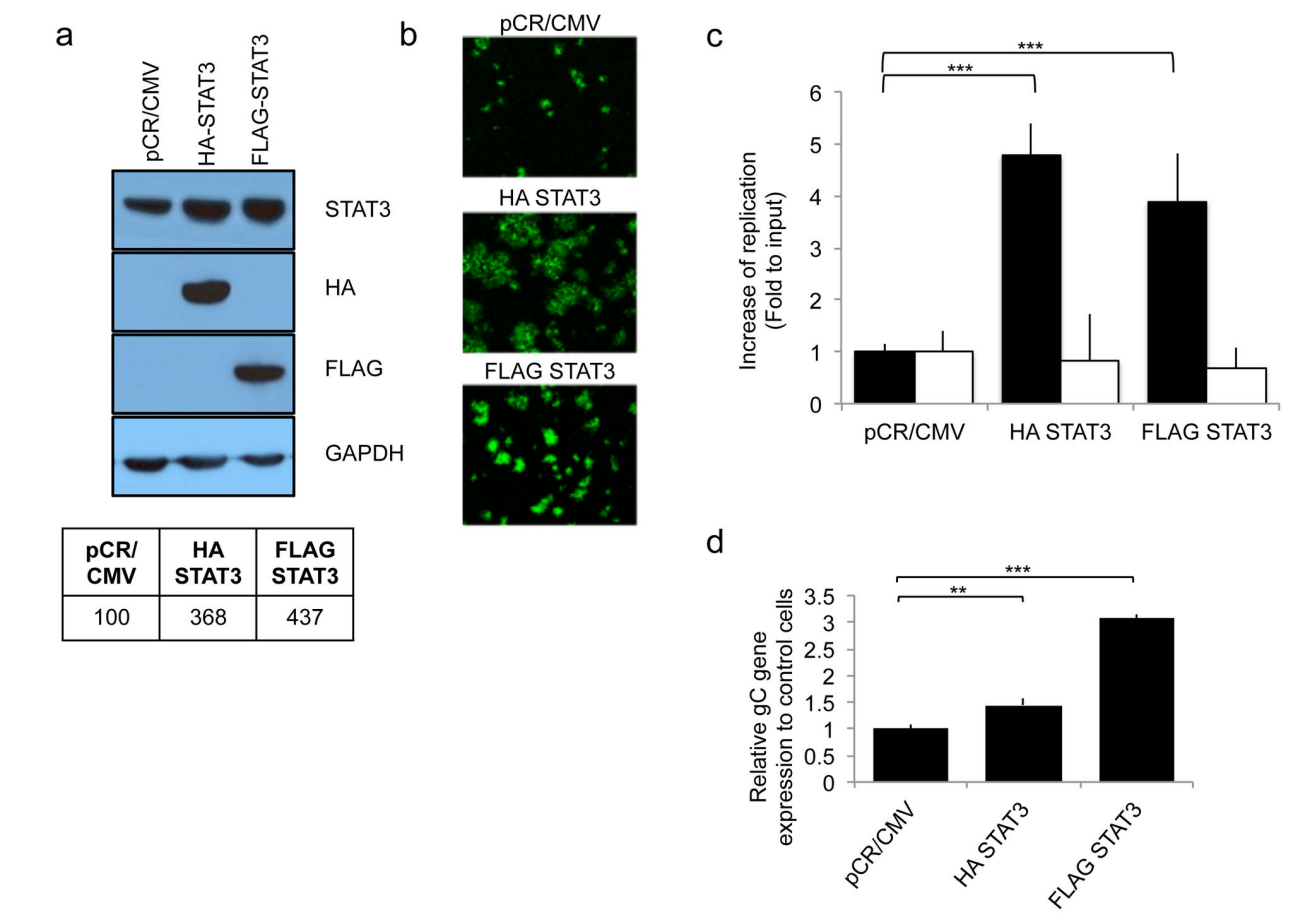
Effect of STAT3 gene expression on oHSV replication in human glioblastoma U251 cells. Control transfected cells (pCR/CMV) were compared to STAT3 over-expressing cells (HA-STAT3 and FLAG-STAT3). a; Western blot analysis of HA-tagged or FLAG-tagged STAT3 in stable transfectants of human U251 glioma cells. Blots of STAT3 and GAPDH were scanned and analyzed by densitometry and are shown in tabular format in the lower part of the panel. The ratios were normalized to pCR/CMV control. b; GFP reporter signal from oHSV (rQNestin34.5) was detected 1 day after oHSV infection (MOI 0.05) of U251 cell lines stably transfected with STAT3. c; *In vitro* viral replication plaque assay. Cells were infected (MOI 0.05) with oHSV (rQNestin34.5) or wild type HSV-1 (F strain) and yields of progeny virus were determined on Vero cells. Black bars represent oHSV. White bars rpresent F strain. Each data point represents the mean of biological triplicates. Error bars indicate standard deviation. ***P < 0.001 (Student’s *t*-test). d; Viral gene expression level of gC was detected using quantitative RT-PCR, 8h following rQNestin34.5 virus infection (MOI 1.0) of U251 cell lines stably transfected with the STAT3 genes. Each data point represents the mean of biological triplicates. Error bars indicate standard deviation. **P < 0.01, ***P < 0.001 (Student’s *t*-test).

**Figure 2:**
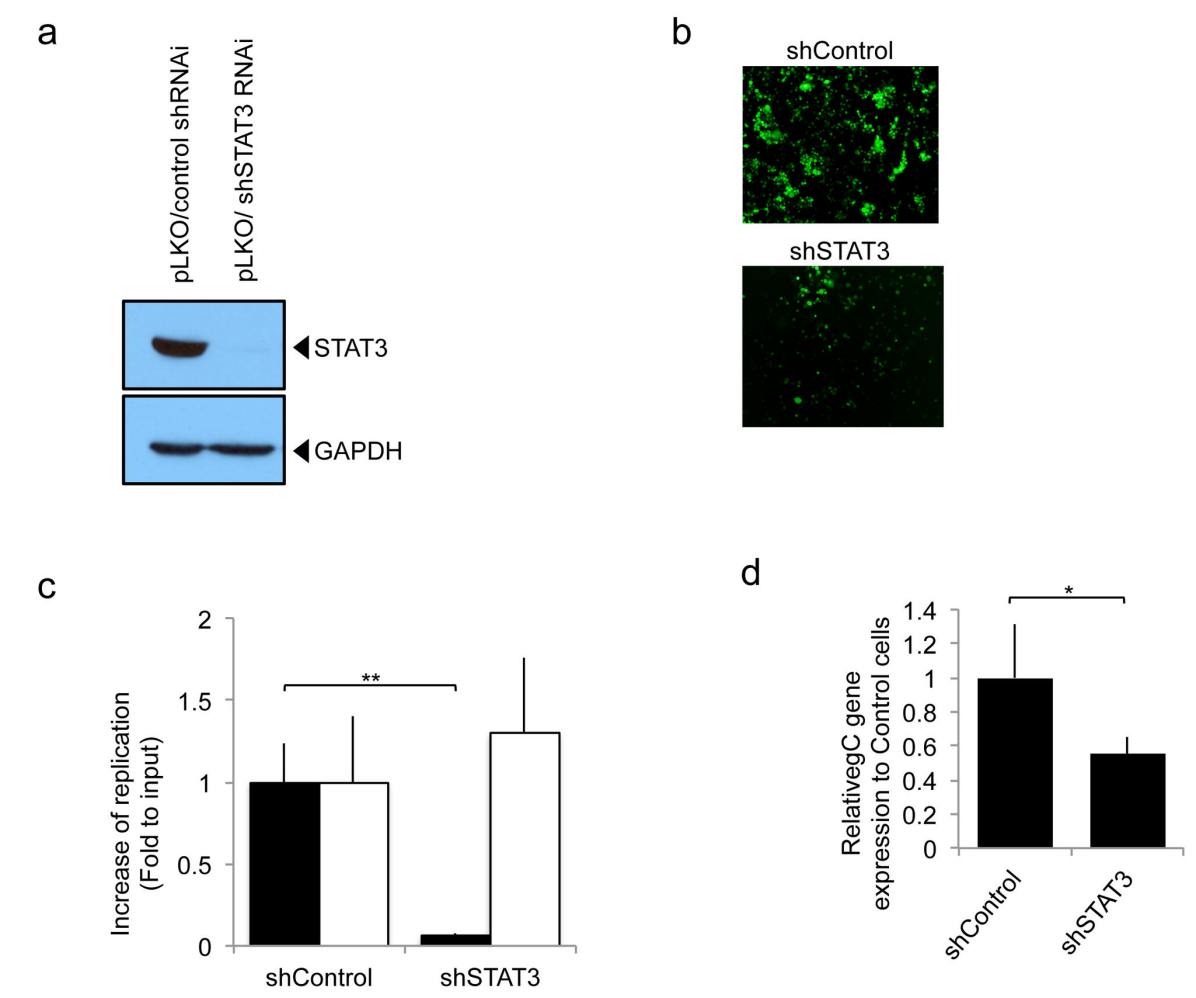
oHSV replication in human glioblastoma U251 cells, stably expressing control shRNA (pLKO/control shRNAi) or STAT3 knock down (pLKO/shSTAT3 RNAi). a; Western blot analysis of STAT3 in the stably transfected control shRNAi or shSTAT3 RNAi. b; GFP reporter signal from oHSV (rQNestin34.5) was detected 1 day after virus infection (MOI 0.05) of U251 cell lines stably knocked down for STAT3 gene expression. c; *In vitro* viral replication plaque assay. Cells were infected (MOI 0.05) with oHSV (rQNestin34.5) or wild type HSV-1 (F strain) and yields of progeny virus were determined on Vero cells. Black bars represent oHSV. White bars represent F strain. Each data point represents the mean of biological triplicates. Error bars indicate standard deviation. **P < 0.01 (Student’s *t*-test). d; Viral gene expression level of gC was detected using quantitative RT-PCR 8h following rQNestin34.5 virus infection (MOI 1.0) of U251 cell lines with stable knock-down of STAT3. Each data point represents the mean of biological triplicates. Error bars indicate standard deviation. *P < 0.05 (Student’s *t*-test).

Recent studies report that STAT3 signaling is a master regulator of mesenchymal transformation of gliomas and that STAT3 modulated genes are highly expressed in the mesenchymal transcriptome of gliomas [12]. We thus asked whether changes in STAT3 expression correlated with changes in oHSV replication. Human U251 glioma cells were stably transfected to express either HA-STAT3 or FLAG-STAT3. This led to three to four times more STAT3, when compared to the basal levels expressed in control pCR/CMV cells (Figure **1a**)**.** Figure **1b** shows that STAT3 over-expressing cells exhibited increased GFP reporter expression and larger and more numerous viral plaques, suggestive of enhanced infectivity/replication, when compared to control pCR/CMV transfected glioma cells. In fact, there was an approximate 30 and 10 fold increase in titers of oHSV in HA STAT3 or FLAG STAT3 expressing glioma cells, respectively, when compared to control pCR/CMV transfectants (Figure **1c** **-black bars**). This was also confirmed by showing that expression of the late viral gene glycoprotein C (gC) was significantly increased in both STAT3 over-expressing transfectants (Figure **1d**). Interestingly, the replication of wild-type, patental HSV1 (F strain) was not affected by STAT3 over-expression (Figure **1c** **-white bars**). Next, we engineered human U251 glioma cell lines with genetic down-regulation of STAT3, using shSTAT3 (Figure **2a**). In contrast to parental cells that expressed basal levels of STAT3, there was a visible reduction in GFP reporter expression and plaque size/number in glioma cells with knocked down STAT3 (shSTAT3) compared to control cells (Figure **2b**). In fact, there was an approximate 100 fold reduction in oHSV titer in glioma cells with stable shRNA knock down of STAT3 compared to control (Figure **2c** **– Black bars**). Comparing the controls of Figure **2b** and Figure **1b**, there was increased GFP fluorescence presumably due to effects of the lentiviral vs. plasmid backbone on baseline cellular homeostasis that increased baseline cellular proliferation in the former vs. the latter (data no shown). Stat3 knock-down also resulted in a significant inhibition of viral gC expression (Figure **2d**). In agreement with the results of Figure **1c**, STAT3 knock-down did not alter the replication of wild-type HSV1 (F strain) (Figure **2c** **- White bars**). Collectively, this data shows that STAT3 expression positively correlates with oHSV replication as well as viral gene expression [12].

### STAT3 gene expression alters oHSV-mediated cytotoxicity of glioma cells

**Figure 3:**
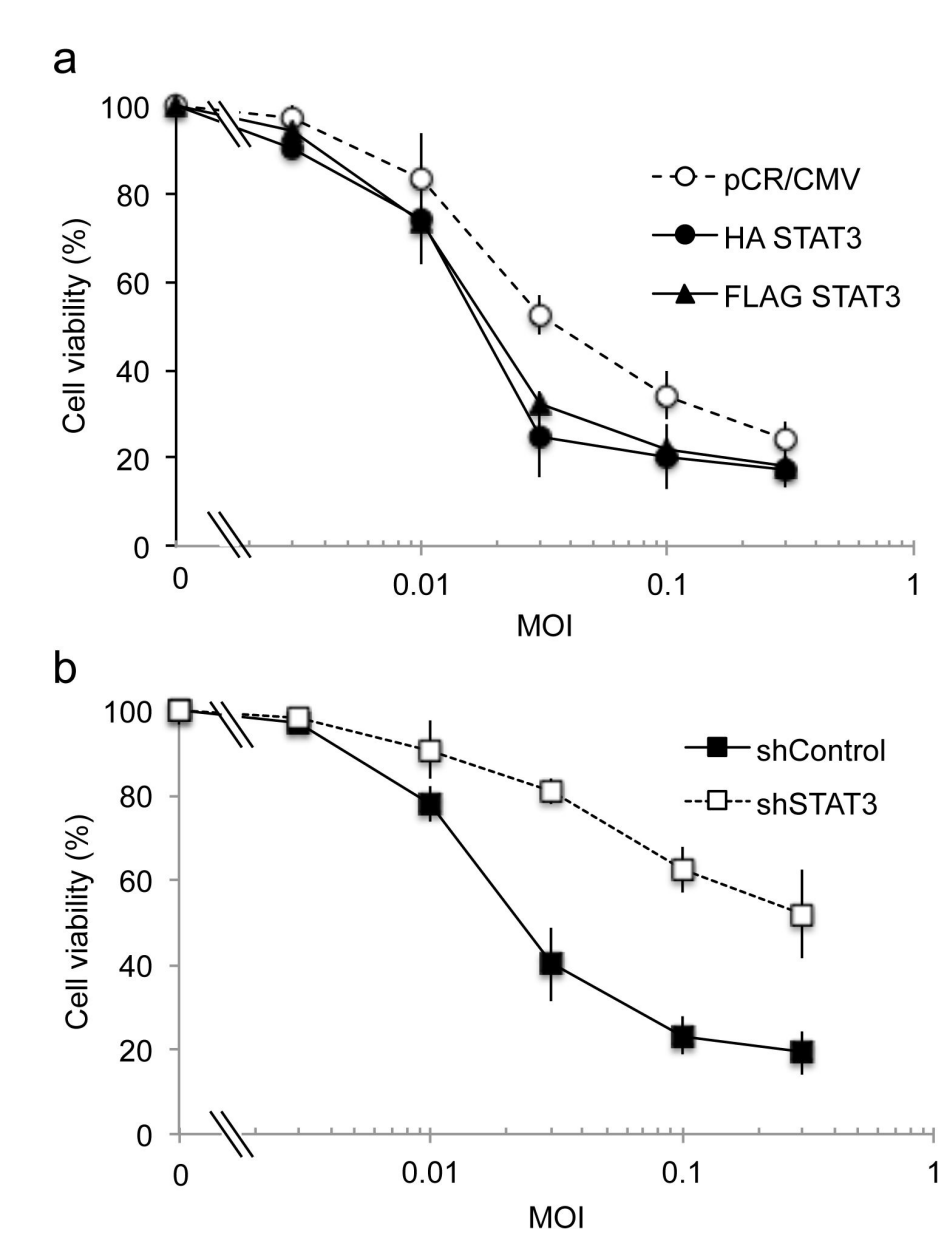
Cytotoxicity of oHSV against glioma cells with altered STAT3 gene expression. a; Control transfected cells (open circle) were compared to STAT3 over-expressing cells HA-STAT3 (closed circle) and FLAG-STAT3 (closed triangle). ED_50_ of pCR/CMV, HA-STAT3, and FLAG-STAT3 were calculated to occur at MOI 0.04, 0.019, and 0.022, respectively. b; lentivirus control transfected cells (closed square) were compared with STAT3 knock down cells (open square). ED_50_ of shControl and shSTAT3 were calculated to be at MOIs 0.025 and >1.0, respectively. Cell viability (measured by MTT) of U251 glioma cells was assayed 3 days after infection of oHSV (rQNestin34.5) at different MOI. Data shown represents the mean ± SD of three replicates for each sample.

Next, we compared the cytotoxicity of oHSV against STAT3 over-expressing glioma cells (Figure **3a**) or glioma cells with STAT3 knock-down by shSTAT3 (Figure **3b**). STAT3 overexpressing cells exhibited cytototoxicity that was measured with an ED_50_ of MOI =0.019 (for HA-STAT3) and 0.022 (for FLAG-STAT3), when compared to that of control pCR/CMV-transfected cells (ED_50_ of MOI of 0.04). Conversely, glioma cells, with STAT3 knock-down exhibited oHSV cytotoxicty at an ED_50_ of >1 MOI, when compared to control shRNA cells (ED_50_ of 0.025 MOI). Therefore, genetic manipulation of STAT3 altered oHSV-mediated cytptoxicity of glioma cells.

### STAT3 reduces type 1 interferon activation in response to oHSV infection

**Figure 4:**
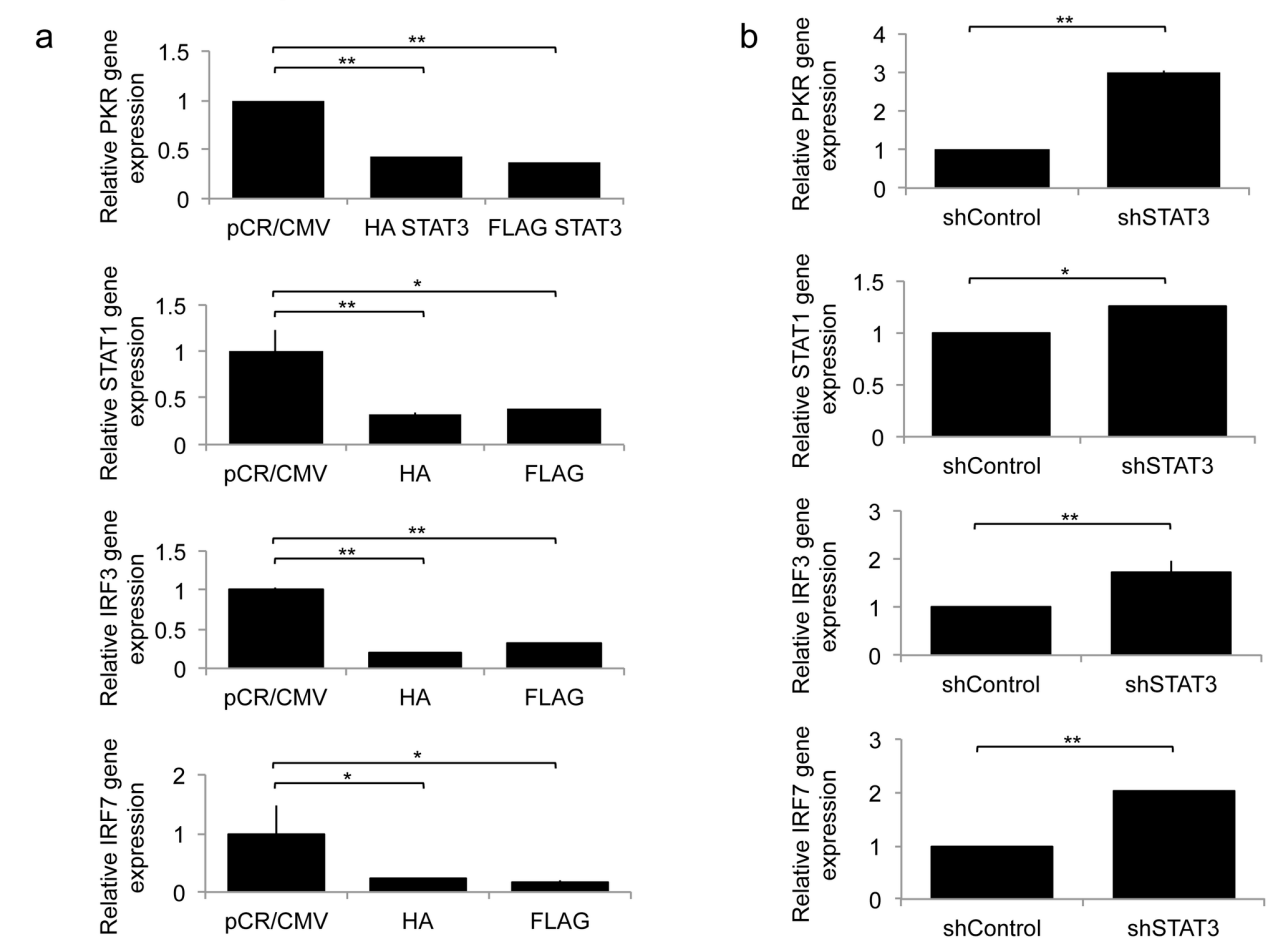
Expression of Type-I IFN responsive genes, PKR, STAT1, IRF3, and IRF7, 8 hours following oHSV (rQNestin34.5) infection (MOI 1.0) in A: U251 cells stably expressing HA-STAT3 or FLAG-STAT3 compared to control pCR/CMV and B: U251 cells stably expressing shSTAT3 RNAi or control. Each data point represents the mean of triplicate samples. Error bars indicate standard deviation. *P < 0.05, **P < 0.01 (Student’s *t*-test).

**Figure 5:**
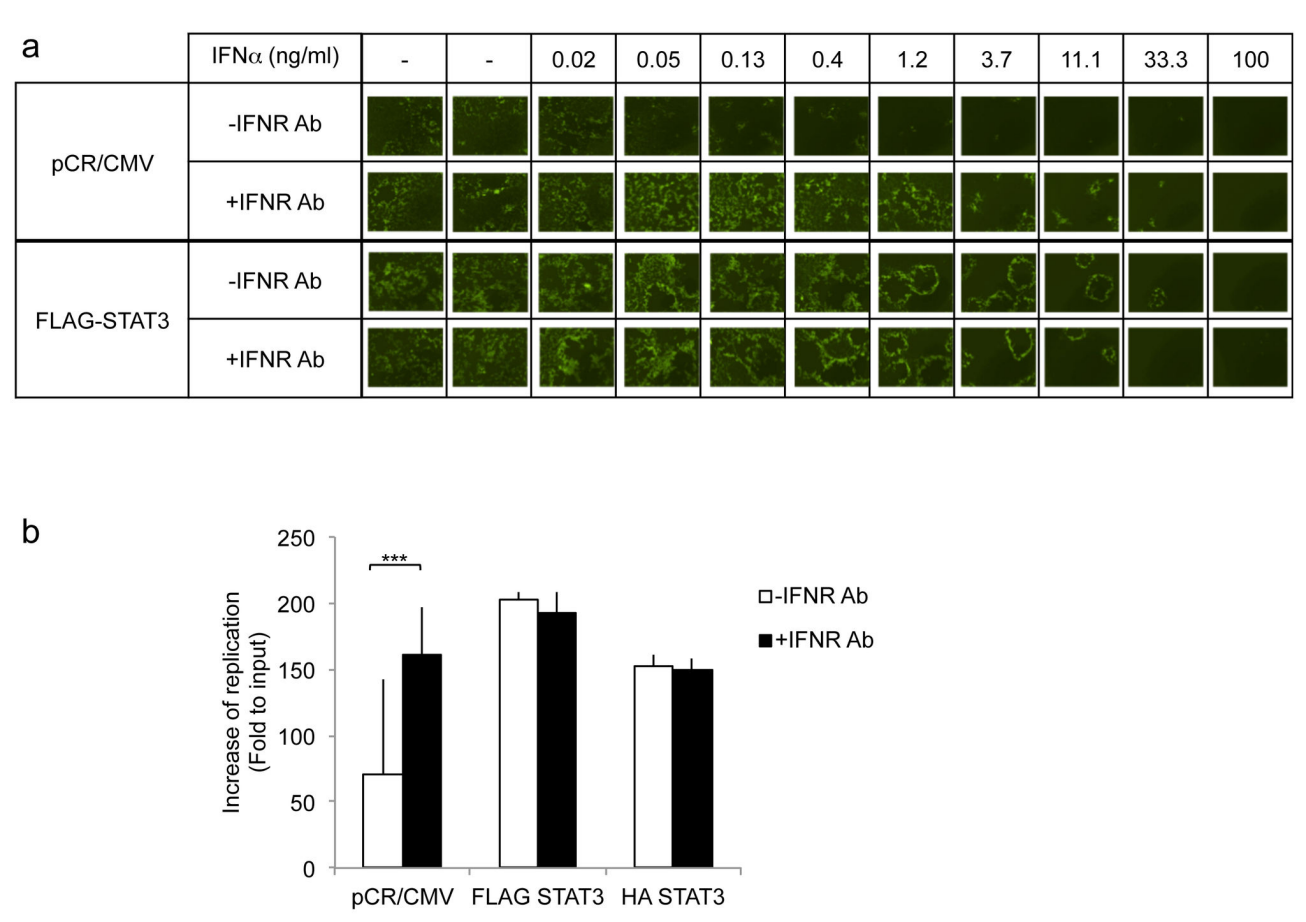
Effect of exogenous IFNalpha with and without IFNalphaR antibody on oHSV replication in cells over-expressing STAT3 compared to control. **A**: Cells were incubated with the indicated concentration of INFalpha in the presence or absence of anti-IFNalpha/beta receptor antibody (IFNR Ab) for 24h, and then infected with oHSV (MOI 0.05). Two days later, GFP expression from oHSV infected cells was visualized. **B**: *In vitro* viral replication assay. U251 transfected cells were treated with (black) or without (white) anti-IFNalpha/betaR (IFNR Ab) antibody for 24 hours. Three days later yields of progeny virus were assayed on Vero cells. Each data point represents the mean of triplicate samples. Error bars indicate standard deviation. ***P < 0.001 (Student’s *t*-test).

We and others have previously shown that oHSV infection leads to a rapid activation of type I interferon signaling pathways [11]. Independently, STAT3 has been shown to decrease type I interferon signaling [13]. We thus asked whether STAT3 activation would reduce type I interferon signaling in the context of an oHSV infection. Figure **4a** shows that, 8 hours after oHSV infection, there was a significant reduction in the expression of interferon response genes (PKR, STAT1, IRF3, and IRF7) in STAT3 expressing cells compared to control cells. Conversely, in glioma cells with stable knock-down of STAT3, there was a significant increase in the expression of PKR, STAT1, IRF3 and IRF7 (Figure **4b**). To further determine if the mechanism behind STAT3’s enhancement of oHSV replication was related to IFN signaling, we utilized antibodies against type I IFN receptors and asked whether they would increase oHSV replication in control vs. STAT3 expressing cells. A representative dose–response experiment showing GFP-positive viral plaques on glioma cells is shown in Figure **5a**. A two day post-infection time is shown because it provided the best visualization of representative plaques. Earlier time points (day 1) showed either small plaques or initially infected cells before formation of plaques. Later time points (day 3) were characterized by more widespread toxicity of cells from the replicating virus, with plaques that became indistinct in borders and co-merged with each other. Figure **5a** shows the expected dose-dependent reduction of GFP-positive plaques after the addition of type 1 IFN to infected control pCR-CMV expressing glioma cell. This reduction was eliminated by the addition of an antibody to the IFNalpha receptor (IFNR). However, in FLAG-STAT3 expressing cells IFNalpha’s reduction of oHSV infection occurred only at very high doses of the cytokine and there was no effect mediated by the antibody. Figure **5b** shows that when type I interferon signaling is blocked, there was a significant increase in oHSV replication in control pCR/CMV cells, but blocking interferon signaling in HA STAT3 or FLAG STAT3 cells did not have an effect on viral replication. In combination, these results thus suggest that the STAT3-mediated reduction in IFN signaling may be the mechanism behind the observed STAT3-mediated increase in oHSV replication in glioma cells.

### Pharmacologic enhancement of STAT3 in glioma cells

**Figure 6:**
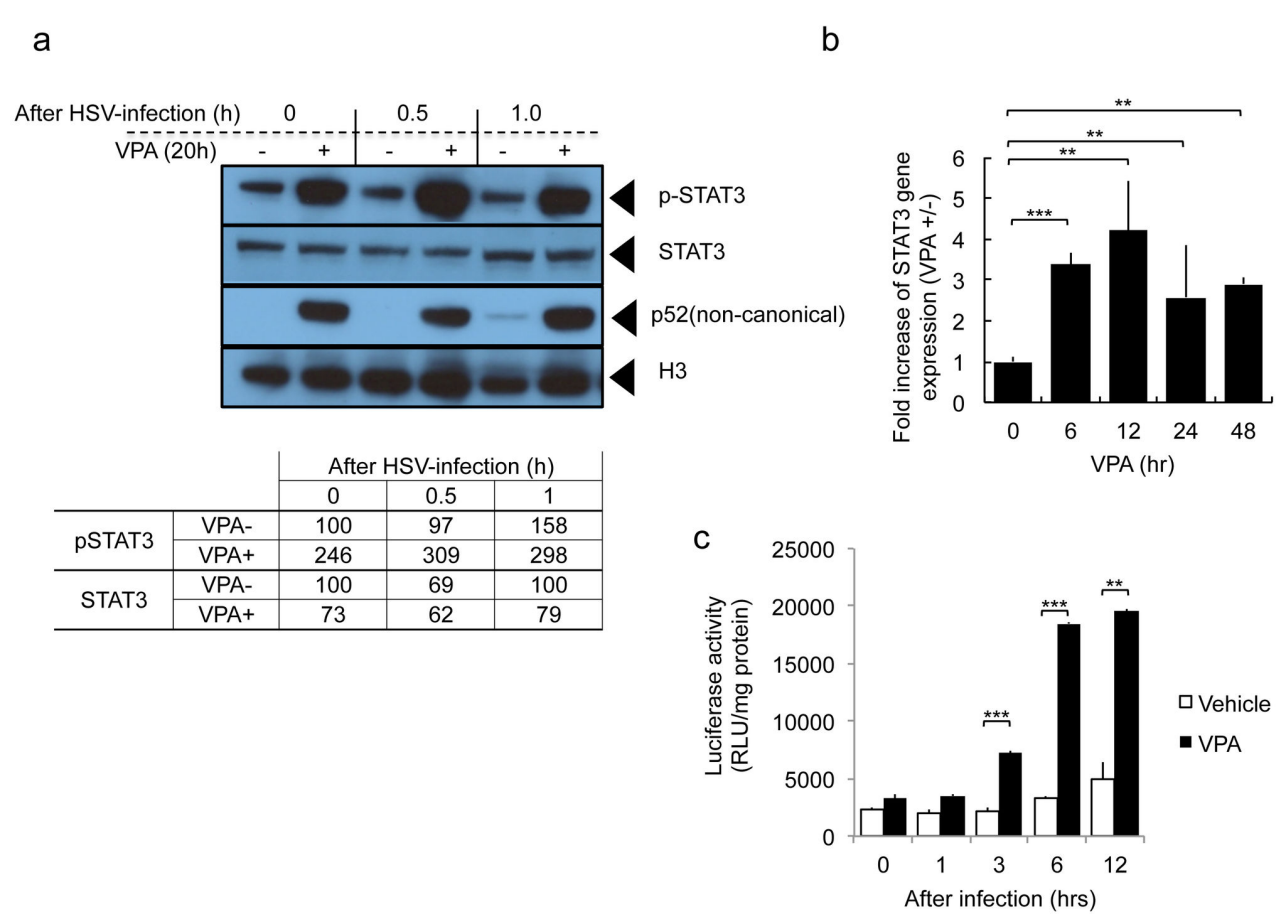
Effect of VPA on STAT3 expression in glioma cells in the presence or absence of oHSV infection. **A**: Expression of nuclear phosho-STAT3 (p-STAT3), total cellular STAT3, activated NF-kappaB (p52), and Histone H3. Twenty hours after incubation with or without 3 mM VPA, U251 cells were infected with oHSV (MOI 1.0). At the indicated times following infection, total cellular or nuclear fractions were collected and analyzed by Western blot. The 0, 0.5 and 1.0 hour (hr) times indicates time after oHSV infection. Before infection, cells were incubated with VPA or vehicle for 20 hours. The results of blot densitometry are also provided in tabular format in the lower part of the panel. **B**: Gene expression level of STAT3 was detected using quantitative RT-PCR at the indicated time points following VPA treatment (3 mM) of U251 cells. Each data point represents the mean of triplicate samples. Error bars indicate standard deviation. **P < 0.01, ***P < 0.001. **C**: Activity of the STAT3 binding promoter was detected following oHSV infection (MOI 1.0) in the presence or absence of VPA. U251 cells were transfected with an expression vector, encoding a STAT3-responsive transcriptional element driving luciferase. After oHSV infection, cells were harvested at the indicated time points and luciferase activity was assayed. White bars = Vehicle, black bars= VPA. Each data point represents the mean of triplicate samples. Error bars indicate standard deviation. **P < 0.01, ***P < 0.001 (Student’s *t*-test).

The preceding data provided a rationale for attempting pharmacologic avenues to increase STAT3 in glioma cells, rendering them more susceptible to oHSV therapy. We have previously shown that valproic acid (VPA) enhances oHSV replication [11]. We thus attempted to determine if VPA altered the levels of STAT3 in glioma cells in the presence or absence of oHSV infection. Figure **6a** shows that VPA treatment increased phosphorylated STAT3 (p-STAT3) nuclear levels in glioma cells, regardless of oHSV infection, but total cellular levels of STAT3 were not affected or perhaps were ven diminished by VPA. This was associated also with an increase in p52 components of NFkB. A time course showed that STAT3 gene expression increased for 12 hours after VPA and remained elevated for at least 48 hours (Figure **6b**). The VPA-mediated increase in STAT3 was likely transcriptional, since it could be replicated in a STAT3 promoter transcriptional activation assay (Figure **6c**). This data thus indicated that VPA could be used to increase phosphorylated STAT3 nuclear expression and this may explain our previously published observation related to VPA’s increase of oHSV replication in glioma cells [11].

### Cytokine enhancement of STAT3 in glioma cells

**Figure 7:**
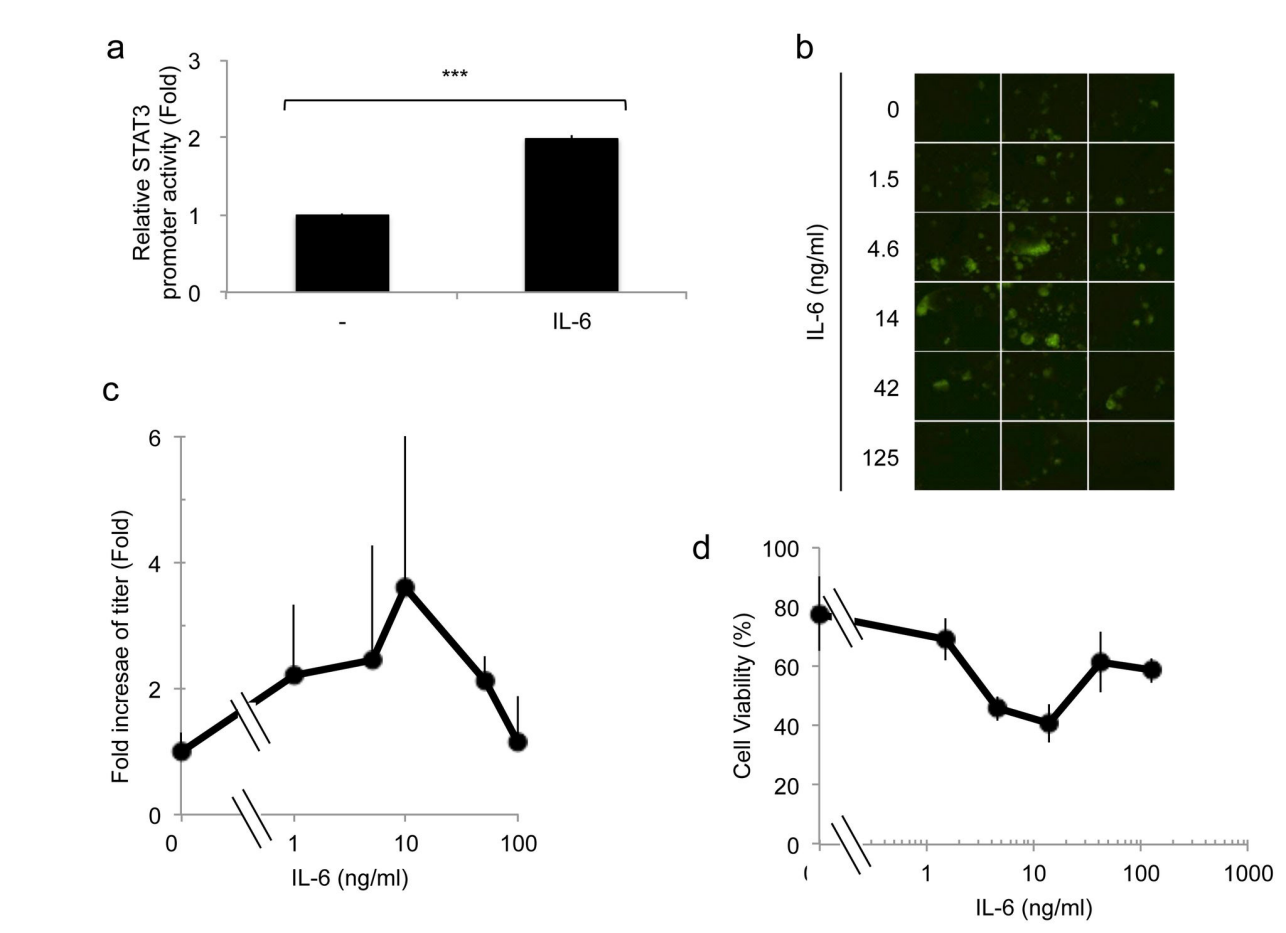
Effect of IL-6 on STAT3 activation and oHSV replication. **A**: Activity of the STAT3 binding promoter was assayed following IL-6 stimulation (10 ng/ml) of U251 cells transfected with an expression vector, encoding a STAT3-responsive transcriptional element driving luciferase. Eight hours following treatment, cells were harvested and luciferase activity was assayed. Each data point represents the mean of biological triplicates. Error bars indicate standard deviation. ***P < 0.001 (Student’s *t*-test). **B**: Human OG02 glioma cells (grown as neurospheres) were infected with oHSV (MOI 0.05) and treated with the indicated concentration of IL-6. One day following infection, GFP activity from oHSV infected cells was visualized. **C**: *In vitro* viral replication assay. OG02 cells were infected with oHSV (MOI 0.05) and treated with the indicated concentration of IL-6. Three days following infection, yields of progeny virus were determined on Vero cells. Each data point represents the mean of biological triplicate samples. Error bars represent the standard deviation. D; Cell viability (measured by MTT) of OG02 glioma cells was assayed 2 days after infection with rQNestin34.5 at different concentration of hIL-6 in the presence of oHSV (MOI 0.05). Data shown represents the mean ± SD of three replicates for each sample.

We also sought to determine if IL6 enhanced oHSV replication. In agreement with published findings, interleukin (IL)-6 treatment of glioma cells increased STAT3 activity (Figure **7a**) [14]. We thus determined whether IL-6 would also led to increased oHSV replication. Figure **7b and 7c** show that there was a IL6 dose-dependent increase in oHSV replication, although at very high doses oHSV replication returned to baseline. Figure **7B** shows fluorescent plaques from GFP-expressing virus after infection of OG02 glioma cells that grow as spheroids (clumps) rather than monolayers. The 3 dimensional nature of the spheroids on a dish tends to render the visualized GFP signal a bit unfocused. There was also an increase in oHSV-mediated cytotoxicity up to a dose of 10ng/ml of IL-6 (Figure **7d**). This data thus agrees with a role for STAT3 in promoting oHSV replication in glioma cells and provides an additional pharmacologic maneuver to increase STAT3 in order to sensitize cells to oHSV.

### Pharmacologic inhibition of STAT3 reduces oHSV replication

**Figure 8:**
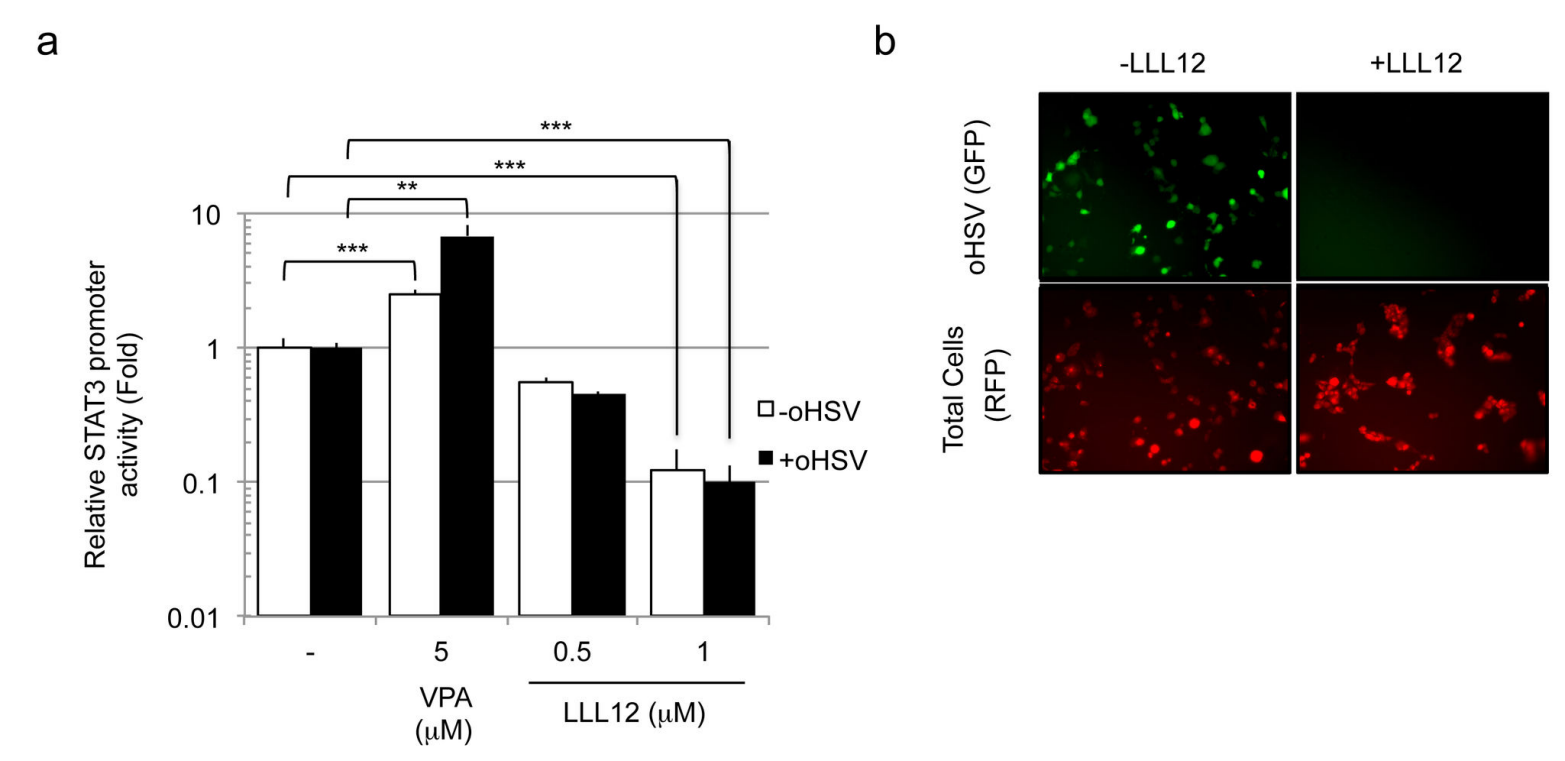
Effect of pharmacologic inhibition of STAT3 on oHSV replication. **A**. U251 cells transfected with an expression vector, encoding a STAT3-responsive transcriptional element driving luciferase were infected with oHSV (MOI 1.0) (Black bars) or control (White bars) following treatment with VPA or the STAT3 inhibitor, LLL12, for 20 hours. Cells were harvested and STAT3 promoter transcriptional activity was assayed. Each data point represents the mean of triplicate samples. Error bars represent the standard deviation. ***P < 0.001, **P < 0.01 **B**: GFP expression from oHSV (rQNestin34.5) infected cells was visualized, 1 day following infection (MOI 0.05) of U251 cell lines pre-incubated with LLL12 for 20h. U251 cells were labeled with the RFP tracker dye. Each data point represents the mean of triplicate samples. Error bars represent the standard deviation.

Finally, we asked whether pharmacologic inhibition of STAT3 reduced oHSV replication. We utilized the small molecule inhibitor of STAT3 phosphorylation at tyrosine 705, LLL12 [15]. Although LLL12 has been reported by several manuscripts to be a STAT3 inhibitor, the original report did not prove this unequivocally [16]. We initially confirmed that LLL12 inhibited STAT3 activation by assaying STAT3-dependent transcriptional luciferase activity (Figure **8a**). In addition, Figure **8a** also confirmed that oHSV infection did not affect STAT3 activity and that VPA increased it as shown before. Next, we determined that oHSV replication in glioma cells was effectively inhibited by 1 microM of LLL12 as assayed by measuring GFP expression from oHSV (Figure **8b**). These results thus provided additional confirmation for a positive role for STAT3 in oHSV replication in glioma cells.

## Discussion

In this report, we show for the first time that STAT3 activation leads to enhanced replication of oHSV in glioma cells. This was also associated with the expected down-regulation of interferon type 1 signaling in response to oHSV infection. Upstream pharmacologic (such as VPA) or cytokine (such as IL-6) activators of STAT3 also enhanced oHSV replication. These results thus imply that tumors with elevated STAT3 may be suitable targets for oHSV. Our results indicate that STAT3 enhances oHSV activity. Whether this depends on overall STAT3 levels or STAT3 phosphorylation will require future experimentation with more specific inhibitors of STAT3 phosphorylation and/or utilizing STAT3 mutants that cannot be phosphorylated.

STAT3 has recently been implicated as an important and significant transcription factor regulating the mesenchymal transformation into aggressive MGs [12]. Its role in glioma stem cell maintenance and self-renewal has been elucidated by many laboratories. In addition, evidence for STAT3 leading to the immunosuppressive milieu of the glioma microenvironment has been mounting [17,18]. One of the immunosuppressive actions of STAT3 in response to viral infections is linked to down-regulation of type 1 interferon signaling [13]. Therefore, the presence of elevated STAT3 activity in human gliomas may be a factor in efficacy of oHSV. In fact, human glioma cells that constitutively express STAT3 significantly enhanced replication of the virus by factors of 10-15, while decreasing STAT3 by shRNA led to the opposite effect. This would suggest that the most aggressive forms of MG (Mesenchymal) may be significant targets for virotherapy based on their STAT3 expression [19].

Our data appears to show that titers of oHSV significantly increased with STAT3 overexpression, but decreased even more significantly when STAT3 was reduced by shRNA. This may imply that basal levels of STAT3 in glioma cells are sufficient to maintain replication of oHSV and that replication needs endogenous STAT3, since reduction can greatly affect the titers of the oHSV. Interestingly, we also determined that this was not true for wild type HSV1, where changes in STAT3 did not appear to alter its titers. The genetic differences between the employed oHSV and F strain are the lack of ICP6 function and one copy of ICP34.5 gene being transcriptionally driven by the cellular nestin promoter rather than by the late viral promoter. This would suggest that one or both of these two functions in the wild-type virus would make its replication independent of STAT3 levels, unlike the oHSV utilized for this study, suggesting a mechanism for rQNestin34.5 tumor selectivity. Perhaps. the altered kinetics of expression of ICP34.5 driven by the cellular nestin promoter in rQNestin34.5 rather than the late HSV1 promoter in wild-type HSV1 alters viral sensitivity to STAT3. However, additional experiments will be required to determine the mechanisms for this difference.

STAT3 expression significantly reduced known factors that belong to IFN-mediated signaling in response to oHSV infection. Although we have not formally proven that the STAT3 mechanism of oHSV-mediated enhancement of viral replication in glioma cells depended on STAT3-mediated reduction of ISG, this remains a very likely mechanism. Other mechanisms that could explain the effect of STAT3 on oHSV replication are less probable or unknown.

Interestingly, we also found that VPA, an HDAC inhibitor, previously shown by us and others to enhance oHSV replication, also increased STAT3 activation in glioma cell and STAT3 reported gene activity. In our previous publication, we had shown that VPA reduced levels of STAT1, PKR and other ISGs [11]. Taken in conjunction, VPA’s mechanism of action through STAT3 activation and ISG reduction would explain the enhanced replication of oHSV, although it is likely that VPA may increase oHSV replication in gliomas though other pathways as well [20]. In our Figure **6**, we show that VPA increases STAT3 promoter transcriptional activity up 12 hours after VPA administration and that this leads to a relative increase in STAT3 gene expression over the first 12 hours after VPA (Figure **6c**) with a subsequent decrease afterwards (Figure 6b). However, this does not translate into an increase in total STAT3 levels by 20 hours after VPA (Figure **6a**). In fact, the most significant effect of VPA on STAT3 appears to be post-translational, since nuclear P-STAT3 levels were dramatically increased 20 hours of VPA (Figure **6a**). We did not analyze total nuclear STAT3 levels to determine if the observed transcriptional increase of STAT3 gene expression resulted in total increased nuclear STAT3. The fact that total cellular STAT3 did not increase 20 hours after VPA may also suggest that there was not sufficient time to alter total cellular STAT3 levels. Interleukin 6 (IL-6), a known inducer of STAT3, also led to increased oHSV replication, further validating the argument that manipulation of STAT3 could be easily achieved by adding either this cytokine or VPA to the oHSV treatment regimen.

In summary, oHSV replication and cytotoxicity can be improved by increasing STAT3 via co-administration of STAT3 activators and by targeting tumors with elevated levels of STAT3. Further experiments in animal models of gliomas should provide an indication of the feasibility of the approach.
